# Digitalisation in the area of alcohol, drug, and tobacco during the COVID-19-pandemic: supporting the drive towards an equitable health? Frida Jonsson

**DOI:** 10.1093/eurpub/ckac129.008

**Published:** 2022-10-25

**Authors:** F Jonsson, R Schimmer

**Affiliations:** 1 Department of Epidemiology and Global Health, Umeå University, Umeå, Sweden; 2 Department of Psychology, Umeå University, Umeå, Sweden

## Abstract

**Background and objectives:**

The COVID-19 pandemic has posed challenges for traditional public health practice. In the area of alcohol, drugs, and tobacco, local and regional actors have largely moved from physical to digital solutions to handle the barriers imposed by the pandemic. To strengthen the knowledge base in the area, this project aimed to explore how the local transition to, and management of, digital solutions within alcohol, drugs, and tobacco prevention might support the policy drive in Sweden towards equity in health.

**Methods:**

This was a qualitative study where 13 local coordinators from 7 municipalities participated. Data were collected through 9 individual and 2 group interviews (semi-structured). The analysis was inductive and followed a thematic analysis approach to identify, analyse, and present patterns (themes) in the data.

**Results:**

Three themes were developed illustrating how the local implementation of digital solutions in the area of alcohol, drugs, doping, and tobacco prevention might support the transition towards equity in health by ‘making time and resources available for development and innovation’, ‘improving the ability to reach and engage with vulnerable groups’, and ‘(re)shape initiatives to act inclusively’.

**Conclusions:**

As illustrated by experiences of the local coordinators, the municipalities seemed to have managed the challenges of the pandemic in a good way. To a large extent, they appeared to have adapted their work to remain operational by transitioning into digital solutions. Considering that the pandemic has been challenging in various ways, the finding of ensuring operations were running should not be underestimated. However, besides being able to largely maintain a “status quo” in a time when traditional modes of working were inadequate or inappropriate, the results illustrated how the municipalities have added numerous (digital) tools to their toolbox for use in the continuing drive towards good and equitable health.

